# Setting the Standard: A Special Focus on Genomic Selection in GENETICS and G3

**DOI:** 10.1534/g3.112.002295

**Published:** 2012-04-01

**Authors:** Dirk-Jan de Koning, Lauren McIntyre

**Affiliations:** * Senior Editor, G3: Genes | Genomes | Genetics, Swedish University of Agricultural Sciences, SE-750 07 Uppsala, Sweden, and; † Senior Editor, GENETICS, University of Florida, Gainesville, Florida 32610

Genomic selection, or genome-wide prediction, was introduced in a landmark publication in GENETICS by Meuwissen *et al.* (2001). The basic premise is to use genotypic information to predict breeding values for particular phenotypes, without specific knowledge of the individual genes contributing to that trait. This article anticipated the widespread availability of affordable, moderate-density SNP genotypes for most livestock and crop species, and developed the next step after QTL mapping and marker assisted selection (MAS).

Since the initial proposal of Meuwissen *et al.* (2001), many alternative approaches for genomic selection have been proposed. The methodology has in large part anticipated data availability, and many articles that introduced new methods employed simulated data or a small scale set of real data. In most cases the data, simulated or actual, were not publicly available, limiting the ability of researchers to compare methods. A notable exception is Crossa *et al.* (2010), where all the data are provided. Currently, the field is awash with interesting proposals. Some attempts at organizing QTL/MAS approaches have been made in Europe, where workshop results have been documented in *BMC Proceedings*. These efforts, while helpful, fail to capture the scholarly discourse that plays out over time in the literature.

In these issues of GENETICS and G3: Genes | Genomes | Genetics, we launch a special focus on genomic selection. GENETICS features a loblolly pine data set and its corresponding analyses (Resende *et al.* 2012); G3 presents a pig data set (Cleveland *et al.* 2012), and a compilation of ten simulated data sets along with the software to simulate more (Hickey and Gorjanc 2012). The goal of these articles is to stimulate discussion in the community, and to provide data for the continuation of the discourse.

We invite additional articles on this topic. Manuscripts that propose new methodologies for genomic selection, as Yang and Templeman (2012), will be expected to include the analysis of at least one common real data set (Crossa *et al.* 2010, Resende *et al.* 2012, Cleveland *et al.* 2012) and be encouraged to use the common simulation platform (Hickey and Gorjanc 2012) to compare the performance of their new method directly to existing methods without extra burden. Authors with new data for which they want to perform genomic selection can select the “best” method to date according to the amassed information. This should free authors from having to apply all the existing methods on their data. The collection of articles (accessed through use of the metadata tag GenPred), will be augmented over time as a result of this structured approach to publishing.

This is not intended to be a special issue or “the final word” on genomic selection. Rather, it is a deliberate “work-in-progress.” The Genetics Society of America journals are a forum for scholarly discussion. We need you—our readers and contributors—to augment analysis of this public data, to add new data, and to debate and improve benchmarking approaches.

There are pitfalls in comparing different approaches on the basis of a limited number of data sets. We hope that comparing different methods against the same panel of real and simulated data spurs thoughtful discussion. We will also consider departures from this structure, if those are well-reasoned. We look forward to submissions of data and novel methods, and discussion about benchmarking.

As your society journals, GENETICS and G3 have an important role to play in fostering discussion and helping define best practice. We believe this special focus is a promising start for genomic selection. Please help us carry this torch by submitting your best work on genomic selection for publication in GENETICS and G3, the peer-edited journals of the Genetics Society of America.
